# The Ottawa Self-Injury Inventory: Evaluation of an assessment measure of nonsuicidal self-injury in an inpatient sample of adolescents

**DOI:** 10.1186/s13034-015-0056-5

**Published:** 2015-07-08

**Authors:** Mary K Nixon, Christine Levesque, Michèle Preyde, John Vanderkooy, Paula F. Cloutier

**Affiliations:** Queen Alexandra Centre for Children’s Health, 2400 Arbutus Rd, Victoria, BC V8N 1V7 Canada; University of Ottawa, 136 Jean-Jacques Lussier, Ottawa, ON K1N 6 N5 Canada; College of Social and Applied Human Sciences, University of Guelph, 50 Stone Road East Mackinnon 138, Guelph, ON N1G 2 W1 Canada; Homewood Health Centre, 150 Delhi St, Guelph, ON N1E 6 K9 Canada; Mental Health Research, Children’s Hospital of Eastern Ontario, 401 Smyth Rd, Ottawa, ON K1H 8 L1 Canada

**Keywords:** Nonsuicidal self-injury, Assessment, Functions, Addictive features, Youth

## Abstract

**Background:**

The Ottawa Self-Injury Inventory (OSI) is a self-report measure that offers a comprehensive assessment of nonsuicidal self-injury (NSSI), including measurement of its functions and addictive features. In a preliminary investigation of self injuring college students who completed the OSI, exploratory analysis revealed four function factors (Internal Emotion Regulation, Social Influence, External Emotion Regulation and Sensation Seeking) and a single Addictive Features factor. Rates of NSSI are particularly high in inpatient psychiatry youth. The OSI can assistin both standardizing assessment regarding functions and potential addictive features and aid case formulation leading to informed treatment planning. This report will describe a confirmatory factor analysis (CFA) of the OSI on youth hospitalized in a psychiatric unit in southwestern Ontario.

**Methods:**

Demographic and self-report data were collected from all youth consecutively admitted to an adolescent in-patient unit who provided consent or assent.

**Results:**

The mean age of the sample was 15.71 years (SD = 1.5) and 76 (81 %) were female. The CFA proved the same four function factors relevant, as in the previous study on college students (*χ*^2^(183) = 231.98, *p* = .008; *χ*^2^/df = 1.27; CFI = .91; RMSEA = .05). The model yielded significant correlations between factors (*r*s = .44-.90, p < .001). Higher NSSI frequency was related to higher scores on each function factor (*r*s = .24-.29, p < .05), except the External Emotion Regulation factor (*r* = .11, *p* > .05). The factor structure of the Addictive Features function was also confirmed (*χ*^2^(14) = 21.96, *p* > .05; *χ*^2^/df = 1.57; CFI = .96; RMSEA = .08). All the items had significant path estimates (.52 to .80). Cronbach’s alpha for the Addictive Features scale was .84 with a mean score of 16.22 (*SD* = 6.90). Higher Addictive Features scores were related to more frequent NSSI (*r* = .48, p < .001).

**Conclusions:**

Results show further support for the OSI as a valid and reliable assessment tool in adolescents, in this case in a clinical setting, where results can inform case conceptualization and treatment planning.

**Electronic supplementary material:**

The online version of this article (doi:10.1186/s13034-015-0056-5) contains supplementary material, which is available to authorized users.

## Background

Early adolescence is the peak period of onset for non suicidal self-injury (NSSI) [1] providing, if detected, an opportunity for early intervention as the youth is at risk of developing a repetitive maladaptive coping strategy. In clinical practice, there are currently no routine standardized self report measures used to inform the understanding and treatment of NSSI despite its high prevalence rates in clinical populations [2, 3]. The majority of NSSI measures remain research tools. Having a measure of NSSI that is valid and clinically useful can inform case conceptualization and treatment planning.

While the clinical interview provides important information and the opportunity to develop a therapeutic alliance, many youth may not share the extent of their NSSI due to shame or difficulty expressing themselves fully in one on one questioning. Many find that self report measures are helpful to share information they would otherwise be reluctant to disclose [4]. In addition, clinicians may not be able to provide as comprehensive questioning specific to NSSI nor necessarily have the time to do so in the first assessment interview. Many aspects of NSSI have been poorly understood in terms of its functions and other characteristics. The Diagnostic and Statistical Manual of Mental Disorders, 5^th^ Edition (DSM-5) [5] has included criteria for NSSI to the section “requiring further study” indicating that NSSI requires more research and proposing that NSSI does not solely exist as a symptom of borderline personality disorder.

Theories regarding the reasons or functions of NSSI have been postulated for several decades with an understanding that NSSI may serve more than one function [6]. Klonsky [7] completed a comprehensive review of theoretical understandings of the functions of NSSI and research to date in the field. Seven main categories of functions of NSSI were derived from this review: affect regulation, self-punishment, antidissociation, interpersonal influence, interpersonal boundaries, sensation-seeking, and anti-suicide. The most commonly endorsed reason for NSSI is affect regulation with the intent to relieve negative affective states such as tension, depression, and/or anger. This category was the most highly endorsed function in a study of hospitalized adolescents where the mean number of endorsed reasons per individual, regardless of category of function, was approximately eight [8]. In a paper entitled “Why do people hurt themselves?”, M. Nock provides an integrated theoretical model of the development and maintenance of NSSI. Distal risk factors such as genetic predisposition to high emotional/cognitive reactivity, intra and interpersonal vulnerability factors, responses to stress and specific NSSI vulnerability factors in the generation of NSSI are illustrated in how they may interact. This model helps to consider those at more risk for development of NSSI and incorporates the role and underpinnings of the potential functions of NSSI [9].

There remains some controversy regarding whether NSSI can become an addictive behaviour despite many youths self reporting this anecdotally and several studies providing evidence of addictive features. In a clinical study of youth with NSSI to study addictive features, Nixon Cloutier and Aggarwal [8], showed that 97.6 % of a clinical sample of 42 repetitive self injuring adolescents endorsed at least three dependence items on a seven-point criteria scale for addictive features of NSSI. This scale was adapted from the Diagnostic Statistical Manual of Mental Disorders IV TR (substance dependence criteria) [10]. Schaub, Holly, Toste, and, Heath [personal communications, 2006], in a university sample of self-injurers, showed that 31 % endorsed at least three of the addictive features using the same seven-item scale. More recently, Moumne, Heath, Schaub, and Nixon [personal communications; 2014] found that of 137 out of 710 high school students surveyed that endorsed lifetime presence of NSSI, 20.4 % reported three or more Additive Features on the OSI addictive features scale. Those with addictive features had higher frequency, more methods and more locations of NSSI. Opposing the concept that NSSI has the potential as an addictive behaviour, Victor, Glenn, and Klonsky [11] found in comparing drug users and self injuring adolescents that cravings occurred primarily while experiencing negative emotions for NSSI with cravings of drug users being higher than that of self injurers.

In reviews of NSSI assessment tools [12, 13] there appears to be significant variability in functions that are measured between assessment tools. Despite the number of self-report measures assessing NSSI functions (e.g., Inventory of Statements About Self-Injury [14], Functional Assessment of Self-Mutilation [15]) none, except the OSI, assess potential addictive features in addition to functions of NSSI. The OSI is a self-report measure that offers a comprehensive assessment of NSSI, including both measurement of its functions and potential addictive features. The inventory was developed based on a comprehensive literature review, clinician feedback and input from adolescent psychiatric inpatients with NSSI. It contains a number of scales including an indication of frequency of recent NSSI thoughts and acts, reasons for starting and reasons for continuing to self injure (i.e., functions), addictive features, level of motivation to stop the behaviour and other characteristics of the nature of NSSI. Youth also respond to questions regarding what has or has not helped in terms of previous treatment (s).

The OSI has been previously validated in a community sample of self-injuring university students [16]. Exploratory factor analyses revealed four function factors (Internal Emotion Regulation, Social Influence, External Emotion Regulation, and Sensation Seeking) and a single Addictive Features factor. Convergent evidence for the functions factors scores was demonstrated through significant correlations with the Functional Assessment of Self-Mutilation measure [15], a known tool for assessing the functions of NSSI. Convergent evidence was also noted for indications of psychological well being, risky behaviours, and context and frequency of NSSI. Convergent evidence for the Addictive Features scores was demonstrated through associations with NSSI frequency, feeling relieved following the act of NSSI, and the inability to resist urges to self injure. The conclusions of this preliminary research were that the OSI is a valid and reliable assessment tool that can be used in both research and clinical settings and that further research is warranted.

The purpose of this report is to describe a confirmatory factor analysis of the functions and addictive scales of the Ottawa Self-Injury Inventory (OSI) on youth hospitalized in a child and adolescent psychiatric inpatient unit in Ontario, Canada. These analyses were performed on data collected for a study on the characteristics of youth who accessed inpatient psychiatric care regarding nonsuicidal self-injury and suicidal behaviour [3]. Comprehensive, accessible and user friendly measures such as the OSI fill a gap in the practice of assessment and offer clinicians a means to objectively assess the behaviour in a standardized fashion.

## Methods

### Subjects

Participants were youth (14 to 18 years old) consecutively admitted between July 2012 and January 2013 to the Child and Adolescent Inpatient unit who gave consent and completed the OSI. The inpatient unit provides in-patient crisis, assessment, stabilization and treatment where the mean length of stay is approximately 5 days.

### Procedures

Youth provided informed consent. Exclusion criteria were an unstable psychiatric condition (e.g., psychosis interfering with the ability to provide informed consent), intellectual disability or pervasive developmental disability which was determined by nursing staff. Consenting youth completed the OSI while in hospital. Research Ethics Board (REB) approval was obtained from the Grand River Hospital, Kitchener-Waterloo, Ontario and the University of Guelph, Guelph, Ontario.

### Measures

The study included self-reported measures of demographics and a standardized measure of NSSI. Data were collected post day two of admission. Youth with a brief one day admission or held overnight were not included.

*Ottawa Self-Injury Inventory* (OSI) [16]: This self report inventory is an in-depth measure of occurrence, frequency, level of motivation to stop, types and functions and potential addictive features of self-injury. The functions of NSSI are endorsed by indicating the degree to which 31 items (e.g., “*to release unbearable tension*”, *“to get care and attention from others”*) correspond with their reasons for engaging in NSSI, ranging from 0, *never a reason*, to 4, *always a reason*). Seven questions were modified from the DSM-IV-TR criteria for substance dependence to incorporate NSSI as opposed to substance use. These were used to assess addictive features (e.g., *“Despite a desire to cut down or control this behaviour, you are unable to do so”*) with a range response options from 0 (*never*) to 4 (*always*) for each addictive feature. The OSI has been shown to be valid and reliable with excellent internal consistency scores of 0.67 to 0.87 in a university sample of young adults [16] and is appropriate for use with clinical samples of adolescents.

### Data analysis

Demographic data was analysed with descriptive statistics using Statistical Package for the Social Sciences (SPSS) Version 21 [17]. Confirmatory factor analysis was used to verify the factor structure of the OSI using AMOS 20 [18]. In order to optimize the sample size, missing values were estimated using Expectation Maximization. None of the items had more than 5 % missing values, indicating that this option was appropriate for use [19].

## Results

In the original sample [3], 322 children and youth were admitted during the study period and assessed by nursing staff for possible inclusion in the study: 102 youth declined to participate or complete the survey, or there were difficulties in obtaining guardian consent, 25 youth were discharged or on pass before they could be asked about the study or before the RA could make contact, 72 did not meet inclusion criteria (48 were considered not appropriate due to psychosis, developmental delay or violent behaviour, 16 were re-admissions, 6 were excluded due to age, one had language difficulties, and one due to extreme fatigue affecting their ability to complete the questionnaires). Ninety-four participants with a lifetime prevalence of NSSI who completed the functions section of the OSI were included in this analysis. Almost half (45.8 %) of the youth reported daily or weekly NSSI and seventy-three percent (*n* = 69) reported co-occurring suicidal ideation and/or behaviour. The mean age was 15.71 (1.5) ranging from 11 to 20 years of age. Eighty-one percent of participants were female, 16 % were male, and one participant was bi-gender. Most youth were attending high school (*n* = 74), four were in middle school, and 7 were in college or university. Approximately three quarters of the sample (*n* = 42) self reported having symptoms of depression.

### Confirmatory factor analysis of function scores

A confirmatory factor analysis (CFA) was conducted to confirm the factor structure of the initial functions of the OSI (“Why did you start to self injure?”). The model was composed of four factors (Internal Emotion Regulation, Social Influence, External Emotion Regulation, and Sensation Seeking). Correlation paths between the factors were allowed. Bootstrapping (5000 samples) was used to manage the presence of multivariate non-normal data within the subsample [20]. The fit of the model was deemed inadequate (*χ*^2^(246) = 402.12, *p* < .001; *χ*^2^/df = 1.64; CFI = .76; RMSEA = .083). Upon further inspection, two items (*to diminish feelings of sexual arousal* and *to get care and attention from other people*) from the *social influence* factor did not have significant path estimates and were therefore removed from the model. In addition, inspection of the modification indexes revealed that one item (*to stop me from thinking about ideas of killing myself*) had significant correlated errors with another item (*to stop me from acting out ideas of killing myself*). This item was also removed from the model. The fit of the final model was deemed satisfactory (*χ*^2^(183) = 231.98, *p* = .008; *χ*^2^/df = 1.27; CFI = .91; RMSEA = .05). All the items in the final model had significant path estimates (standardized factor loadings are presented in Table 1). This model also yielded significant correlations between each factors (see Table 2). Greater NSSI frequency was related to higher scores on each function factor (*r*s = .24–.29, *p* < .05), except for the External Emotion Regulation factor (*r* = .11, *p* > .05).

**Table 1:** Standardized factor loadings and descriptive statistics for NSSI function factors

Motivations	Internal Emotion Regulation	Social Influence	External Emotion Regulation	Sensation Seeking
To produce a sense of being real when I feel numb and “unreal”	.64			
To relieve feelings of sadness or feeling “down”	.63			
To distract me from unpleasant memories	.62			
To punish myself	.60			
To stop feeling alone and empty	.56			
To experience physical pain in one area, when the other pain I feel is unbearable	.56			
To stop me from acting out ideas of killing myself	.50			
To stop my parents from being angry at me		.56		
To stop people from expecting so much from me		.55		
To change my body image and/or appearance		.53		
To show others how hurt or damaged I am		.50		
To avoid getting in trouble for something I did		.46		
To get out of doing something that I don’t want to do		.38		
To belong to a group		.29		
To release frustration			.89	
To release anger			.80	
To release unbearable tension.			.62	
To experience a “high” like a drug high				.71
To provide a sense of excitement that feels exhilarating				.69
For sexual excitement				.31
To prove to myself how much I can take				.26
*α*	.78	.66	.82	.53
Mean scores (*SD*)	17.78 (7.11)	5.47 (4.93)	8.62 (3.49)	3.69 (3.39)

**Table 2:** Intercorrelations between the function factors

	1	2	3	4
1. Internal Emotion Regulation	-	.68***	.90***	-.76***
2. Social Influence		-	.44***	-.87***
3. External Emotion Regulation			-	-.59***
4. Sensation Seeking				-

*Note*. *** *p* < .001

### Confirmatory factor analysis of addictive features

Ninety one of ninety four participants completed the Addictive Features items. The same analytic strategy as described previously for the function items was conducted on the seven Addictive Features items of the OSI. The fit of the model was deemed satisfactory (*χ*^2^(14) = 21.96, *p* > .05; *χ*^2^/df = 1.57; CFI = .96; RMSEA = .08). All the items had significant path estimates, ranging between .52 and .80 (standardized factor loadings are presented in Table 3). Cronbach’s alpha for the Addictive Features scale was .84 with a mean score of 16.22 (*SD* = 6.90).

**Table 3:** Standardized factor loadings and descriptive statistics for NSSI Addictive Features

Items	Addictive Features
The self-injurious behaviour occurs more often than intended?	.64
The severity in which the self-injurious behaviour occurs has increased (e.g., deeper cuts, more extensive parts of your body)?	.80
If the self-injurious behaviour produced an effect when started, you now need to self-injure more frequently or with greater intensity to produce the same effect?	.74
This behaviour or thinking about it consumes a significant amount of your time (e.g., planning and thinking about it, collecting and hiding sharp \objects, doing it and recovering from it)?	.60
Despite a desire to cut down or control this behaviour, you are unable to do so?	.68
You continue this behaviour despite recognizing that it is harmful to you physically and/or emotionally?	.59
Important social, family, academic or recreational activities are given up or reduced because of this behaviour?	.52
*α*	.84
Mean scores (SD)	16.22 (6.90)

Higher Addictive Features scores were related to more frequent NSSI (*r* = .48, *p* < .001). In addition, no significant correlation was found between the Addictive Features factor and feeling of physical pain when self-injuring (*r* = .05, *p* > .05). Lastly, significant positive correlations between the Addictive Features factor and each of the obtained function factors of the OSI were obtained (*r*s = .30–.44, *p* < .01).

## Discussion

The current study provides additional support for the psychometric properties of the OSI’s functions and Addictive Features scales in a clinical sample of adolescents. The original factor structure obtained in a university sample [16] was confirmed. The four-factor model (Internal Emotion Regulation, Social Influence, External Emotion Regulation, and Sensation Seeking) of NSSI functions and the single Addictive Features factor were replicated in this clinical sample, with few exceptions. Within the Internal Emotion Regulation factor, the item *“to stop me from thinking about ideas of killing myself”* had significant correlated errors with the item *“to stop me from acting out ideas of killing myself”*. This is not a surprising finding as the two items are connected when there is active planning of a suicide attempt, in that experiencing suicidal ideation commonly precedes the act of suicide. Under the Social Influence factor there were two items that did not have significant path estimates (i.e., did not relate significantly to their factor), namely, “*to diminish feelings of sexual arousal”* and “*to get care and attention from other people”.* It is unclear why this would be, however, these items may be under-reported or less commonly reported in adolescent inpatients. Inpatient samples have typically higher rates and frequency of NSSI [21] and are likely to have functions endorsed related to managing symptoms associated with major mental health disorders such as mood and anxiety problems. Additional research is recommended to investigate this further.

Convergent evidence was found for scores on both functions and Addictive Features on the OSI through significant correlations with theoretical and empirical constructs. Specifically, greater NSSI frequency was related to higher scores on each function factor, except for the External Emotion Regulation factor. This finding further supports the notion that frequent NSSI can be both negatively (Internal Emotional Regulation) and positively (Sensation Seeking) reinforcing in a clinical sample as previously found in a non clinical population [16]. The mean score in this clinical sample was double that obtained in the university sample (16.22 vs 8.05) indicating that the measure is sensitive enough to detect differences between samples. These findings indicate that clinical samples might have more addictive features of NSSI than community samples however further research is required.

An interesting finding is that Social Influence as a function factor was correlated with frequency of NSSI in this clinical sample while this was not the case in Martin and colleagues [16], where the population was somewhat older and also community based. There may be several reasons for this finding. Firstly, adolescents as opposed to young adults are expected to have fewer and less developed coping strategies [22]. Second, the adolescent period is particularly stressful in regards to interpersonal issues, more specifically the impact of peer influence and peer victimization including online bullying [23]. Thirdly, clinical samples typically have greater frequency of NSSI than non-clinical samples and triggers or reasons for NSSI such as social influence factors are likely to also be reinforces of the behaviour leading to more frequent NSSI.

There are several study limitations that should be mentioned. First and foremost, the sample size limits generalizability of the results and research should replicate these findings with large samples. Second, there were fewer males than females who participated in the study. However, the gender proportions obtained are representative of the ratio of females to males admitted to adolescent inpatient care [2, 8]. Further research on males in clinical populations who engage in NSSI is required. Third, as this was a secondary analysis of survey data obtained from a clinical sample, we were unable to fully explore convergent and discriminant validity with the data being limited to what was obtained in the original sample [3].

## Conclusions

This current study provides additional support for the psychometric properties of the OSI’s functions and Addictive Features scales. Further research on larger clinical and community samples is warranted. Clinicians can use a self report method that is comprehensive and validated in an adolescent clinical population. In a recent study of adolescents with self harm [4], the investigators found that self report was able to detect previously undetected NSSI in a clinical setting, suggesting that while self report questionnaires do not replace clinical assessment, they may enhance detection rates in youth. While the purpose of this study was to confirm a preliminary factor analysis, further research clinically in terms of enhancing detection is indicated.

Several recent studies [24, 25] have reviewed treatment interventions that show promise in youth with NSSI. As Brent and colleagues [25] emphasize in their summary, results for both suicide attempts and NSSI should be reported separately. An assessment tool such as the OSI could give both baseline and outcome information specifically on NSSI and its associated functions and features. Ougrin and colleagues [24] in their systematic review and meta analysis of therapeutic interventions for suicide attempts and self harm in adolescents indicate that that largest effect sizes are for dialectical behavior therapy (DBT), cognitive behavioural therapy (CBT) and mentalization based therapy (MBT), but that no modality has had its efficacy independently replicated. They highlight that research is lacking in indentifying variables that are most important to match youths with NSSI and their families to interventions that may have the most benefit. With the ability to assess functions based on four factors (Internal Emotion Regulation, Social Influence, External Emotion Regulation, and Sensation Seeking) and the extent of Addictive Features, the OSI may assist in selecting more specific treatment modalities. For example, for those with the Internal Emotional Regulation function most highly endorsed, assessment for mood and anxiety disorders would be important and the components of DBT and or CBT may be most indicated whereas those with the Social Influence function most highly endorsed and related attachment issues MBT may be more beneficial. For those with significant Addictive Features endorsed, managing treatment expectations and using a harm reduction approach with motivational interviewing may be most helpful. More research in these areas is needed as the treatment of NSSI in youth continues to lack standardized assessment and knowledge about what might be the most effective treatments depending on the nature of the behavior [26].

### Measure

The OSI can be downloaded free of charge if used for public institutions and for research purposes at http://www.insync-group.ca/publications/OSI_clinical_October_20051.pdf (Additional file 1).

## Additional file

Additional file 1:
**The Ottawa Self-Injury Inventory.**

## Abbreviations

NSSI: Nonsuicidal self-injury
DSM: Diagnostic and Statistical Manual of Mental Disorders
OSI: Ottawa Self-Injury Inventory
CFA: Confirmatory factor analysis
SD: Standard deviation
RMSEA: Root Mean Square Error of Approximation
