# Supplementary material for: The Ottawa Self-Injury Inventory: Evaluation of an assessment measure of nonsuicidal self-injury in an inpatient sample of adolescents
Source: Child Adolesc Psychiatry Ment Health. 2015 Jul 8;9:26. doi: 10.1186/s13034-015-0056-5 (PMC4495629; doi:10.1186/s13034-015-0056-5)
Supplement: Additional file 1: — The Ottawa Self-Injury Inventory. [file 13034_2015_56_MOESM1_ESM.pptx]

## Slide 1
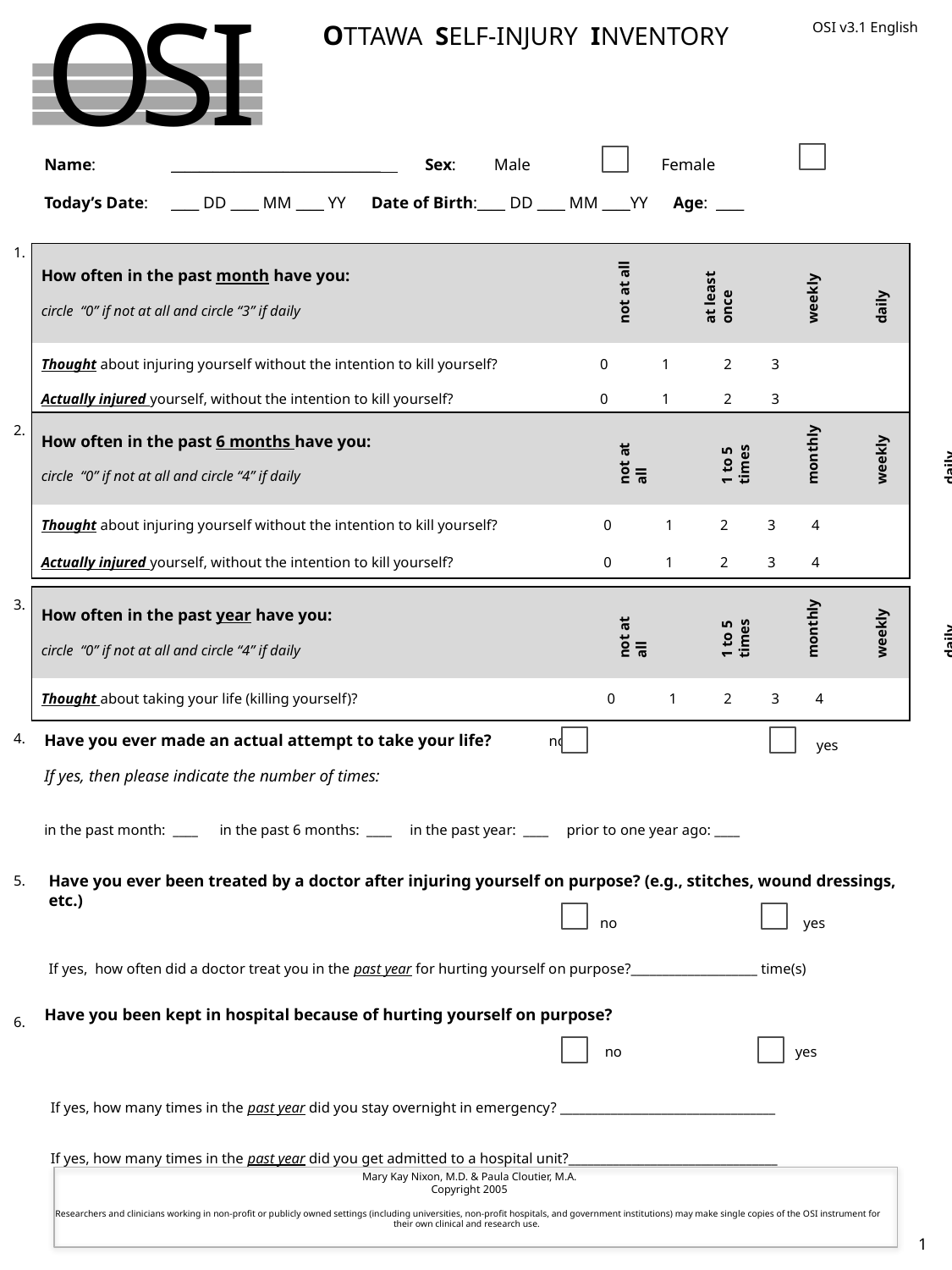

Name:	______________________________ 	Sex: Male Female
Today’s Date:	____ DD ____ MM ____ YY Date of Birth:____ DD ____ MM ____YY Age: ____
1.
| How often in the past month have you: circle “0” if not at all and circle “3” if daily | not at all         at least once       weekly     daily |
| --- | --- |
| Thought about injuring yourself without the intention to kill yourself? | 0 1 2 3 |
| Actually injured yourself, without the intention to kill yourself? | 0 1 2 3 |
| How often in the past 6 months have you: circle “0” if not at all and circle “4” if daily | not at all         1 to 5 times       monthly       weekly       daily |
| --- | --- |
| Thought about injuring yourself without the intention to kill yourself? | 0 1 2 3 4 |
| Actually injured yourself, without the intention to kill yourself? | 0 1 2 3 4 |
2.
| How often in the past year have you: circle “0” if not at all and circle “4” if daily | not at all         1 to 5 times       monthly       weekly       daily |
| --- | --- |
| Thought about taking your life (killing yourself)? | 0 1 2 3 4 |
3.
4.
Have you ever made an actual attempt to take your life? no
If yes, then please indicate the number of times:
in the past month: ____ in the past 6 months: ____ in the past year: ____ prior to one year ago: ____
yes
Have you ever been treated by a doctor after injuring yourself on purpose? (e.g., stitches, wound dressings, etc.)
If yes, how often did a doctor treat you in the past year for hurting yourself on purpose?____________________ time(s)
5.
no
yes
Have you been kept in hospital because of hurting yourself on purpose?
6.
no
yes
If yes, how many times in the past year did you stay overnight in emergency? __________________________________
If yes, how many times in the past year did you get admitted to a hospital unit?_________________________________
1

## Slide 2
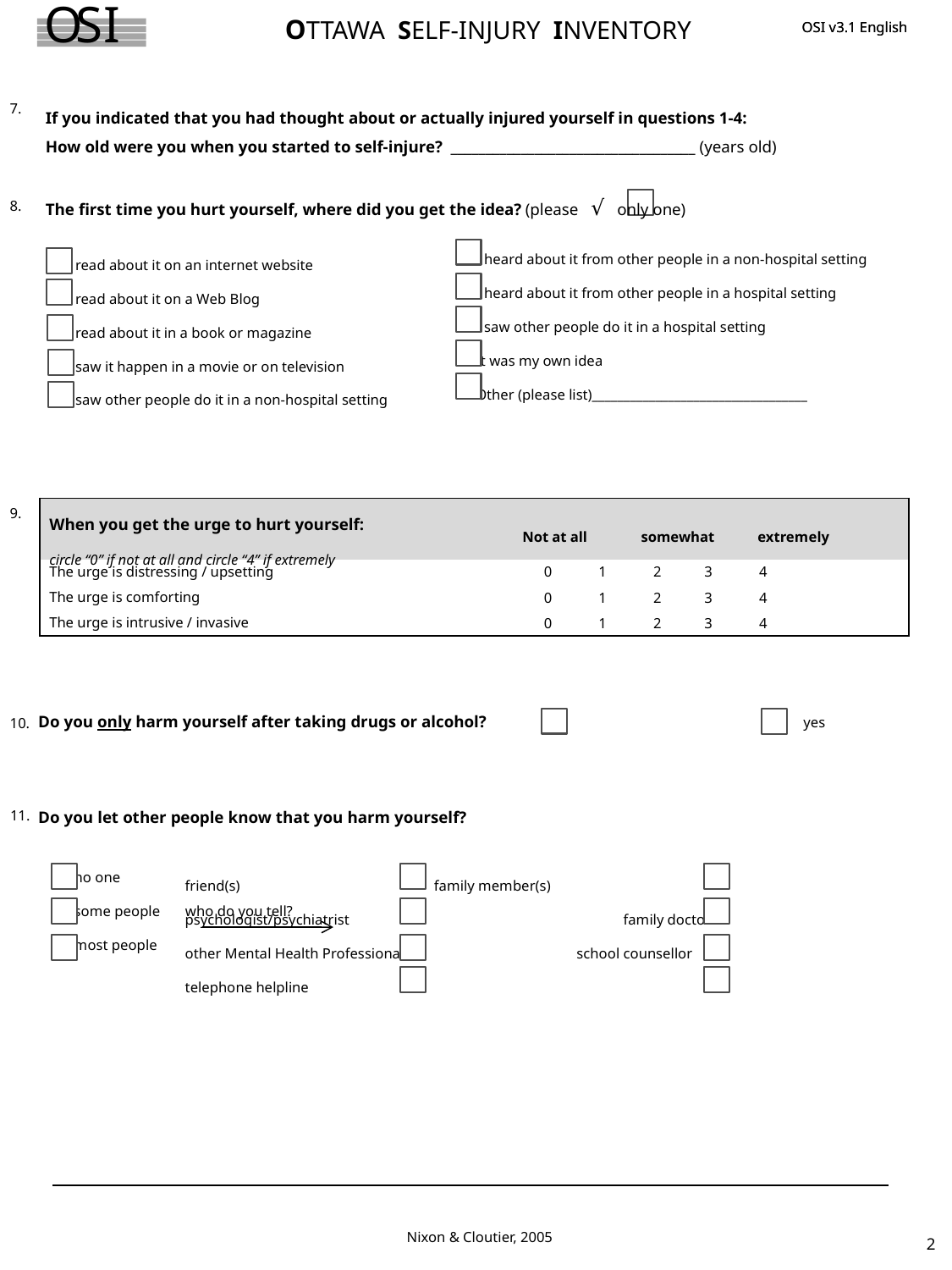

If you indicated that you had thought about or actually injured yourself in questions 1-4:
How old were you when you started to self-injure? ___________________________________ (years old)
The first time you hurt yourself, where did you get the idea? (please √ only one)
7.
8.
 I heard about it from other people in a non-hospital setting
 I heard about it from other people in a hospital setting
 I saw other people do it in a hospital setting
 It was my own idea
 Other (please list)__________________________________
 I read about it on an internet website
 I read about it on a Web Blog
 I read about it in a book or magazine
 I saw it happen in a movie or on television
 I saw other people do it in a non-hospital setting
9.
| When you get the urge to hurt yourself: circle “0” if not at all and circle “4” if extremely | Not at all somewhat extremely |
| --- | --- |
| The urge is distressing / upsetting | 0 1 2 3 4 |
| The urge is comforting | 0 1 2 3 4 |
| The urge is intrusive / invasive | 0 1 2 3 4 |
Do you only harm yourself after taking drugs or alcohol? no 		 yes
Do you let other people know that you harm yourself?
 no one
 some people who do you tell?
 most people
10.
11.
 friend(s) 	 family member(s)
 psychologist/psychiatrist		 family doctor
 other Mental Health Professional	 school counsellor
 telephone helpline					 other (specify)
2

## Slide 3
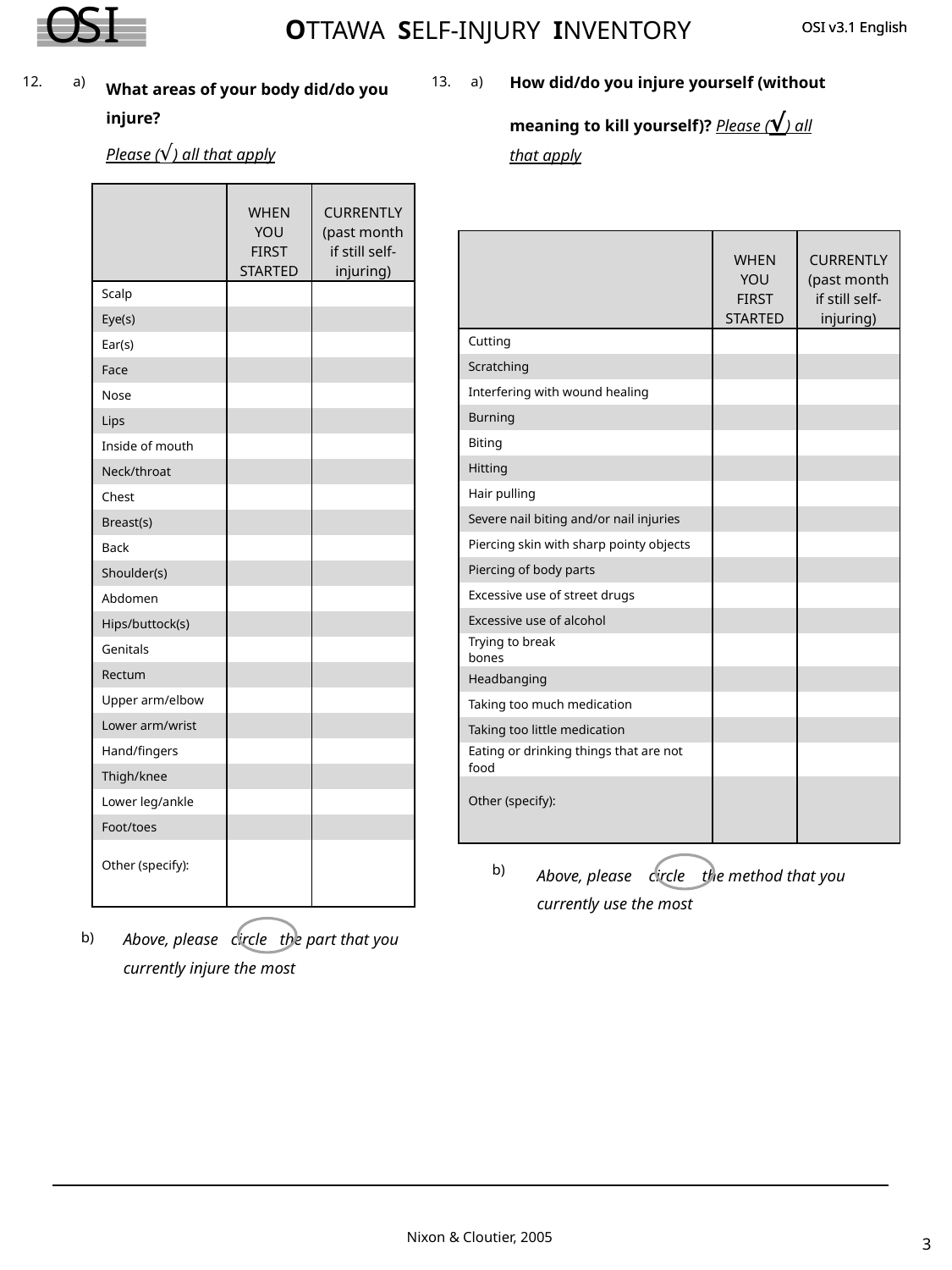

How did/do you injure yourself (without meaning to kill yourself)? Please (√) all that apply
What areas of your body did/do you injure?
Please (√) all that apply
a)
13.
12.
a)
| | WHEN YOU FIRST STARTED | CURRENTLY (past month if still self-injuring) |
| --- | --- | --- |
| Scalp | | |
| Eye(s) | | |
| Ear(s) | | |
| Face | | |
| Nose | | |
| Lips | | |
| Inside of mouth | | |
| Neck/throat | | |
| Chest | | |
| Breast(s) | | |
| Back | | |
| Shoulder(s) | | |
| Abdomen | | |
| Hips/buttock(s) | | |
| Genitals | | |
| Rectum | | |
| Upper arm/elbow | | |
| Lower arm/wrist | | |
| Hand/fingers | | |
| Thigh/knee | | |
| Lower leg/ankle | | |
| Foot/toes | | |
| Other (specify): | | |
| | WHEN YOU FIRST STARTED | CURRENTLY (past month if still self-injuring) |
| --- | --- | --- |
| Cutting | | |
| Scratching | | |
| Interfering with wound healing | | |
| Burning | | |
| Biting | | |
| Hitting | | |
| Hair pulling | | |
| Severe nail biting and/or nail injuries | | |
| Piercing skin with sharp pointy objects | | |
| Piercing of body parts | | |
| Excessive use of street drugs | | |
| Excessive use of alcohol | | |
| Trying to break bones | | |
| Headbanging | | |
| Taking too much medication | | |
| Taking too little medication | | |
| Eating or drinking things that are not food | | |
| Other (specify): | | |
Above, please circle the method that you currently use the most
b)
Above, please circle the part that you currently injure the most
b)
3

## Slide 4
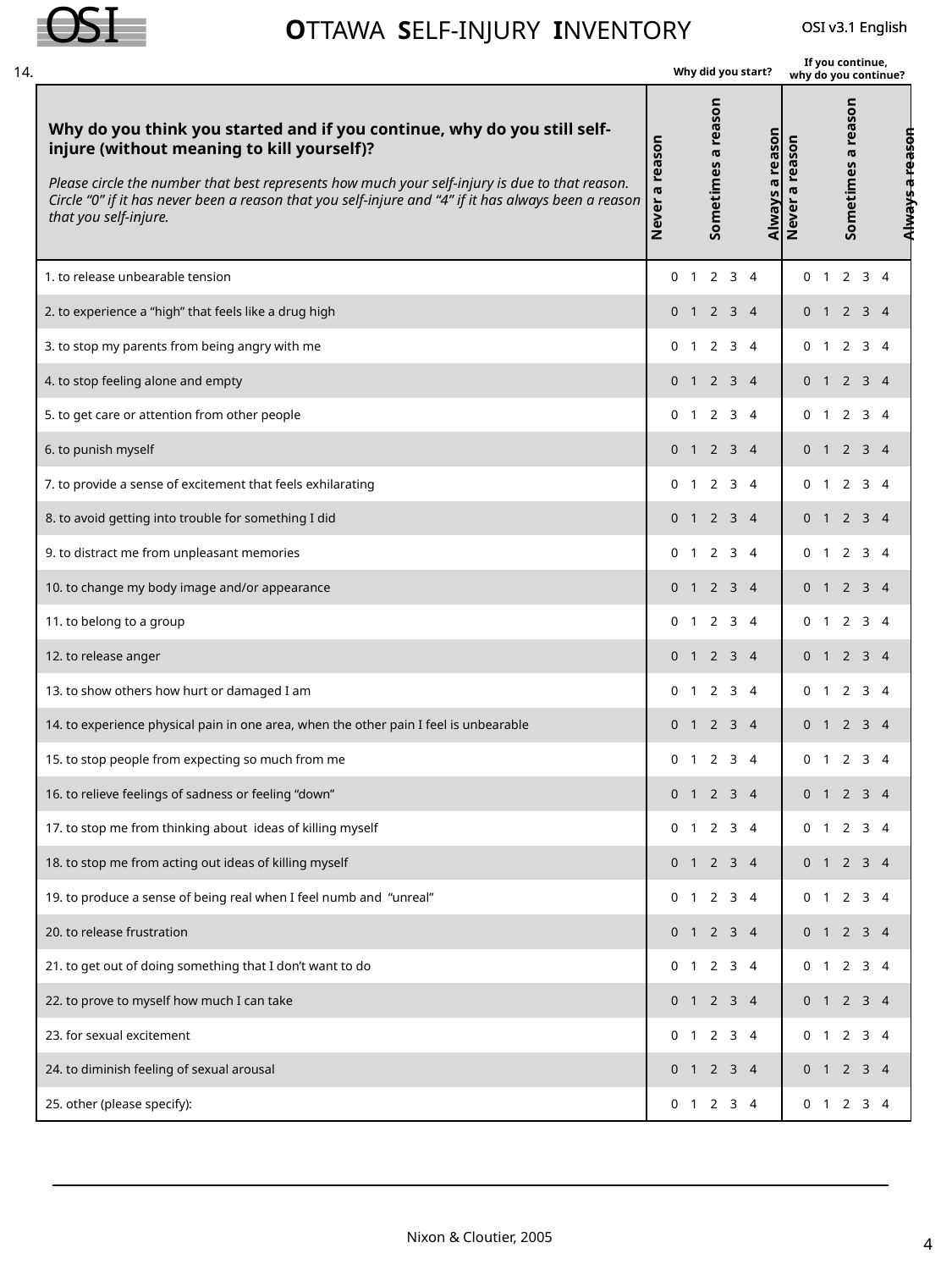

If you continue,
why do you continue?
14.
Why did you start?
| | Never a reason Sometimes a reason Always a reason | Never a reason Sometimes a reason Always a reason |
| --- | --- | --- |
| 1. to release unbearable tension | 0 1 2 3 4 | 0 1 2 3 4 |
| 2. to experience a “high” that feels like a drug high | 0 1 2 3 4 | 0 1 2 3 4 |
| 3. to stop my parents from being angry with me | 0 1 2 3 4 | 0 1 2 3 4 |
| 4. to stop feeling alone and empty | 0 1 2 3 4 | 0 1 2 3 4 |
| 5. to get care or attention from other people | 0 1 2 3 4 | 0 1 2 3 4 |
| 6. to punish myself | 0 1 2 3 4 | 0 1 2 3 4 |
| 7. to provide a sense of excitement that feels exhilarating | 0 1 2 3 4 | 0 1 2 3 4 |
| 8. to avoid getting into trouble for something I did | 0 1 2 3 4 | 0 1 2 3 4 |
| 9. to distract me from unpleasant memories | 0 1 2 3 4 | 0 1 2 3 4 |
| 10. to change my body image and/or appearance | 0 1 2 3 4 | 0 1 2 3 4 |
| 11. to belong to a group | 0 1 2 3 4 | 0 1 2 3 4 |
| 12. to release anger | 0 1 2 3 4 | 0 1 2 3 4 |
| 13. to show others how hurt or damaged I am | 0 1 2 3 4 | 0 1 2 3 4 |
| 14. to experience physical pain in one area, when the other pain I feel is unbearable | 0 1 2 3 4 | 0 1 2 3 4 |
| 15. to stop people from expecting so much from me | 0 1 2 3 4 | 0 1 2 3 4 |
| 16. to relieve feelings of sadness or feeling “down” | 0 1 2 3 4 | 0 1 2 3 4 |
| 17. to stop me from thinking about ideas of killing myself | 0 1 2 3 4 | 0 1 2 3 4 |
| 18. to stop me from acting out ideas of killing myself | 0 1 2 3 4 | 0 1 2 3 4 |
| 19. to produce a sense of being real when I feel numb and “unreal” | 0 1 2 3 4 | 0 1 2 3 4 |
| 20. to release frustration | 0 1 2 3 4 | 0 1 2 3 4 |
| 21. to get out of doing something that I don’t want to do | 0 1 2 3 4 | 0 1 2 3 4 |
| 22. to prove to myself how much I can take | 0 1 2 3 4 | 0 1 2 3 4 |
| 23. for sexual excitement | 0 1 2 3 4 | 0 1 2 3 4 |
| 24. to diminish feeling of sexual arousal | 0 1 2 3 4 | 0 1 2 3 4 |
| 25. other (please specify): | 0 1 2 3 4 | 0 1 2 3 4 |
Why do you think you started and if you continue, why do you still self-injure (without meaning to kill yourself)?
Please circle the number that best represents how much your self-injury is due to that reason. Circle “0” if it has never been a reason that you self-injure and “4” if it has always been a reason that you self-injure.
4

## Slide 5
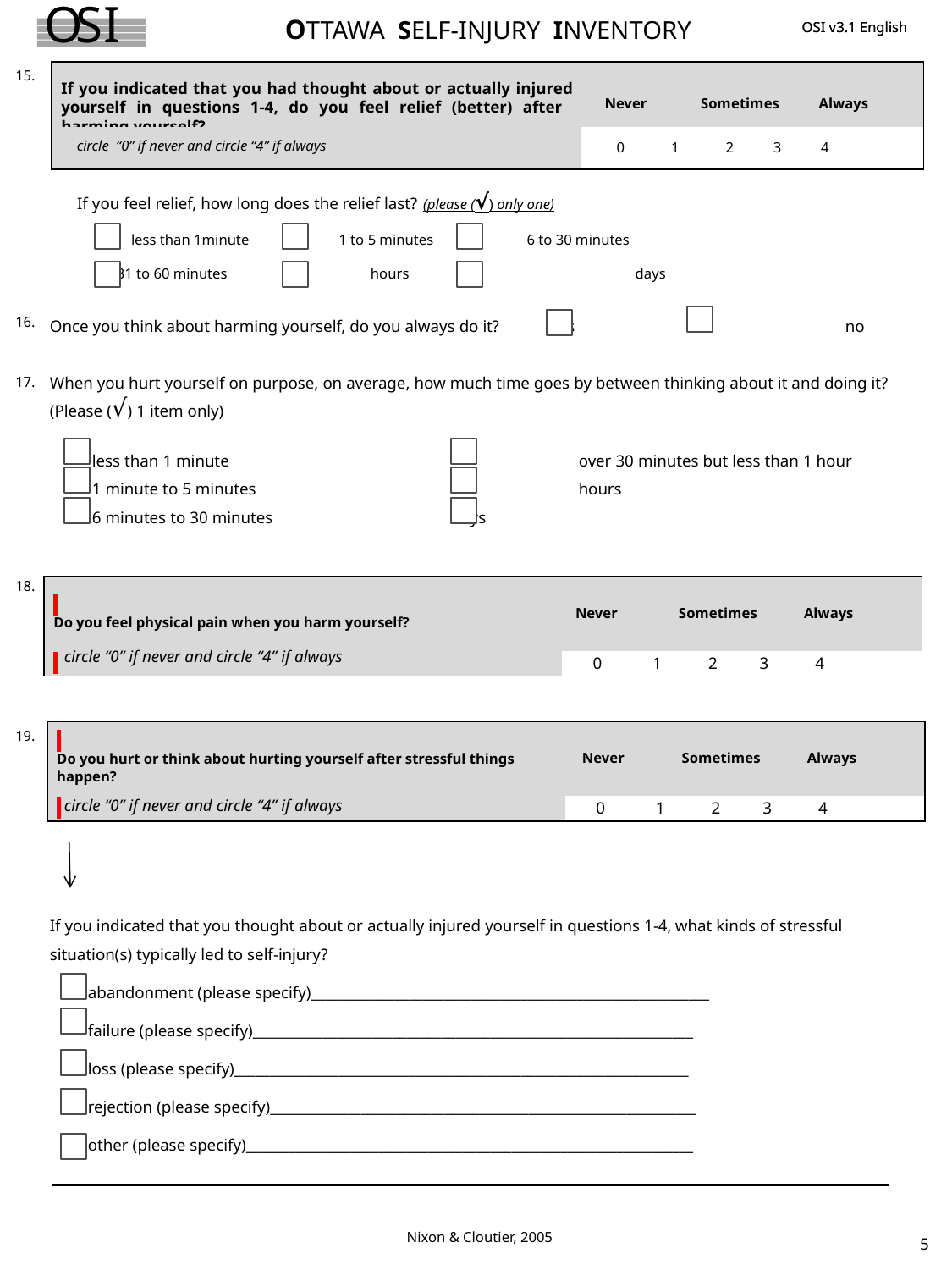

15.
| If you indicated that you had thought about or actually injured yourself in questions 1-4, do you feel relief (better) after harming yourself? | Never Sometimes Always |
| --- | --- |
| | 0 1 2 3 4 |
If you feel relief, how long does the relief last? (please (√) only one)
 less than 1minute 1 to 5 minutes 6 to 30 minutes
 31 to 60 minutes 	 hours	 	 days
 circle “0” if never and circle “4” if always
16.
Once you think about harming yourself, do you always do it? yes 		 no
17.
When you hurt yourself on purpose, on average, how much time goes by between thinking about it and doing it? (Please (√) 1 item only)
 less than 1 minute		 over 30 minutes but less than 1 hour
 1 minute to 5 minutes		 hours
 6 minutes to 30 minutes	 days
18.
| Do you feel physical pain when you harm yourself? | Never Sometimes Always |
| --- | --- |
| | 0 1 2 3 4 |
circle “0” if never and circle “4” if always
19.
| Do you hurt or think about hurting yourself after stressful things happen? | Never Sometimes Always |
| --- | --- |
| | 0 1 2 3 4 |
circle “0” if never and circle “4” if always
If you indicated that you thought about or actually injured yourself in questions 1-4, what kinds of stressful situation(s) typically led to self-injury?
 abandonment (please specify)_________________________________________________________
 failure (please specify)_______________________________________________________________
 loss (please specify)_________________________________________________________________
 rejection (please specify)_____________________________________________________________
 other (please specify)________________________________________________________________
5

## Slide 6
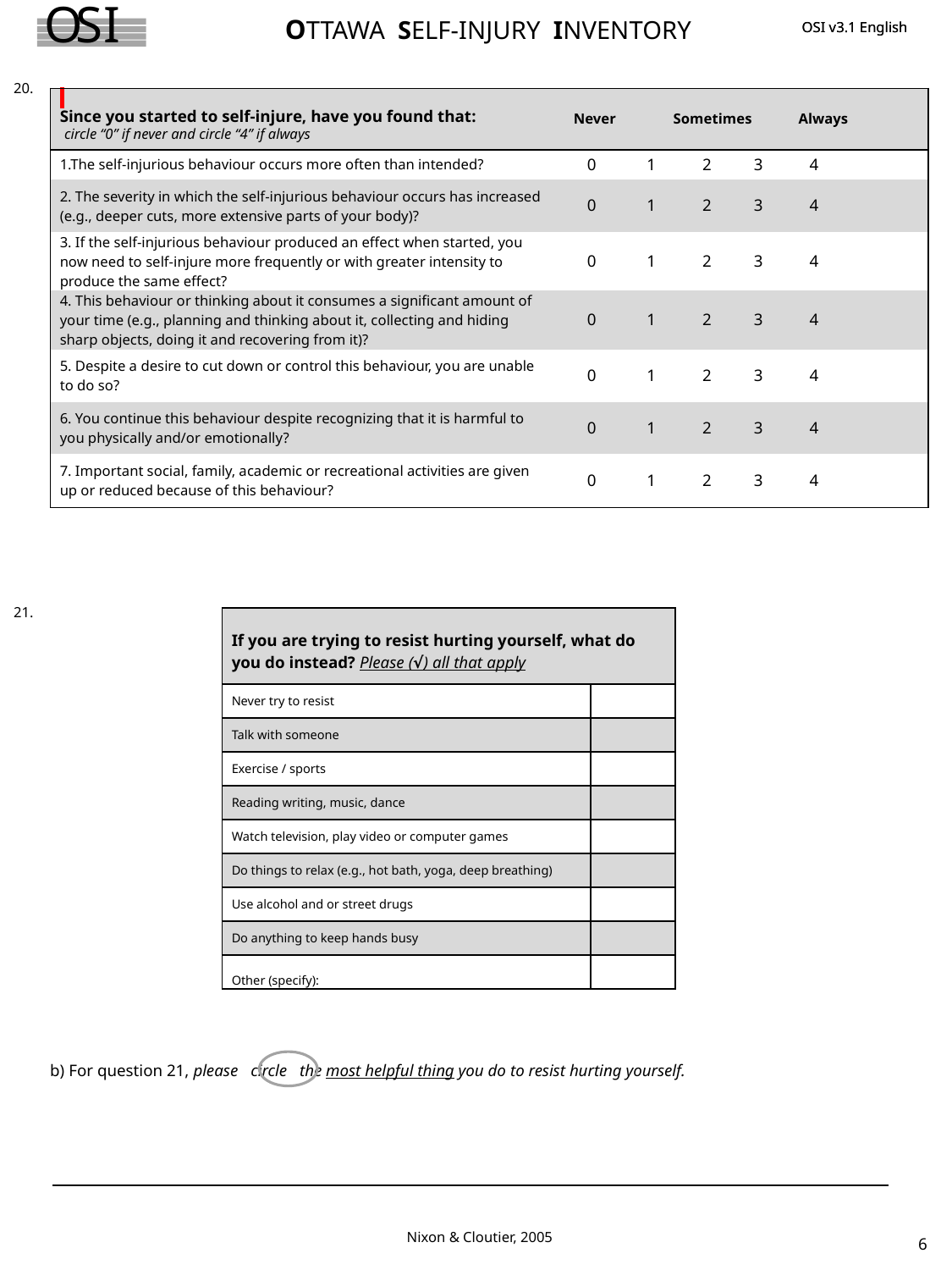

20.
| Since you started to self-injure, have you found that: | Never Sometimes Always |
| --- | --- |
| 1.The self-injurious behaviour occurs more often than intended? | 0 1 2 3 4 |
| 2. The severity in which the self-injurious behaviour occurs has increased (e.g., deeper cuts, more extensive parts of your body)? | 0 1 2 3 4 |
| 3. If the self-injurious behaviour produced an effect when started, you now need to self-injure more frequently or with greater intensity to produce the same effect? | 0 1 2 3 4 |
| 4. This behaviour or thinking about it consumes a significant amount of your time (e.g., planning and thinking about it, collecting and hiding sharp objects, doing it and recovering from it)? | 0 1 2 3 4 |
| 5. Despite a desire to cut down or control this behaviour, you are unable to do so? | 0 1 2 3 4 |
| 6. You continue this behaviour despite recognizing that it is harmful to you physically and/or emotionally? | 0 1 2 3 4 |
| 7. Important social, family, academic or recreational activities are given up or reduced because of this behaviour? | 0 1 2 3 4 |
circle “0” if never and circle “4” if always
21.
| If you are trying to resist hurting yourself, what do you do instead? Please (√) all that apply | |
| --- | --- |
| Never try to resist | |
| Talk with someone | |
| Exercise / sports | |
| Reading writing, music, dance | |
| Watch television, play video or computer games | |
| Do things to relax (e.g., hot bath, yoga, deep breathing) | |
| Use alcohol and or street drugs | |
| Do anything to keep hands busy | |
| Other (specify): | |
b) For question 21, please circle the most helpful thing you do to resist hurting yourself.
6

## Slide 7
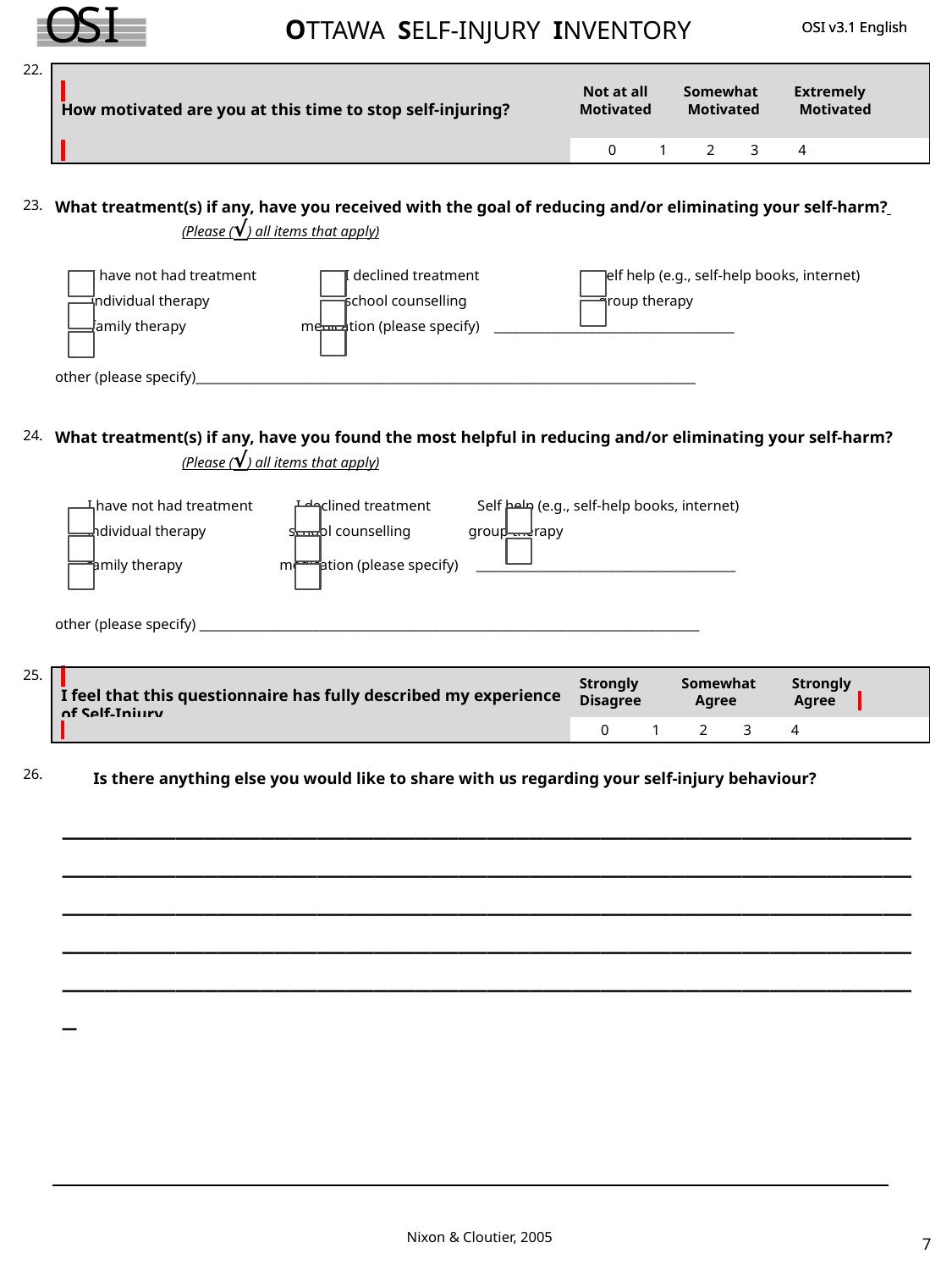

22.
| How motivated are you at this time to stop self-injuring? | Not at all Somewhat Extremely Motivated Motivated Motivated |
| --- | --- |
| | 0 1 2 3 4 |
23.
What treatment(s) if any, have you received with the goal of reducing and/or eliminating your self-harm?
	(Please (√) all items that apply)
 I have not had treatment	 I declined treatment	 Self help (e.g., self-help books, internet)
 individual therapy	 school counselling	 group therapy
 family therapy medication (please specify) ______________________________________
other (please specify)_______________________________________________________________________________
What treatment(s) if any, have you found the most helpful in reducing and/or eliminating your self-harm?
	(Please (√) all items that apply)
 I have not had treatment I declined treatment Self help (e.g., self-help books, internet)
 individual therapy school counselling group therapy
 family therapy medication (please specify) _________________________________________
other (please specify) _______________________________________________________________________________
24.
25.
| I feel that this questionnaire has fully described my experience of Self-Injury | Strongly Somewhat Strongly Disagree Agree Agree |
| --- | --- |
| | 0 1 2 3 4 |
26.
Is there anything else you would like to share with us regarding your self-injury behaviour?
_____________________________________________________________________________________________________________________________________________________________________________________________________________________________________________________________________________________________________________
7
